# Pain Management Strategies in Osteoarthritis

**DOI:** 10.3390/biomedicines12040805

**Published:** 2024-04-04

**Authors:** Luca Farinelli, Michele Riccio, Antonio Gigante, Francesco De Francesco

**Affiliations:** 1 Clinical Orthopaedics, Department of Clinical and Molecular Sciences, Università Politecnica delle Marche, 60121 Ancona, Italy; luca.farinelli@ospedaliriuniti.marche.it (L.F.); a.p.gigante@staff.univpm.it (A.G.); 2 Department of Reconstructive Surgery and Hand Surgery, Azienda Ospedaliera Universitaria delle Marche, 60126 Ancona, Italy; michele.riccio@ospedaliriuniti.marche.it

**Keywords:** osteoarthritis, pain, therapy, management, review

## Abstract

Pain is the major symptom of osteoarthritis (OA) and is an important factor in strategies to manage this disease. However, the current standard of care does not provide satisfactory pain relief for many patients. The pathophysiology of OA is complex, and its presentation as a clinical syndrome is associated with the pathologies of multiple joint tissues. Treatment options are generally classified as pharmacologic, nonpharmacologic, surgical, and complementary and/or alternative, typically used in combination to achieve optimal results. The goals of treatment are the alleviation of symptoms and improvement in functional status. Several studies are exploring various directions for OA pain management, including tissue regeneration techniques, personalized medicine, and targeted drug therapies. The aim of the present narrative review is to extensively describe all the treatments available in the current practice, further describing the most important innovative therapies. Advancements in understanding the molecular and genetic aspects of osteoarthritis may lead to more effective and tailored treatment approaches in the future.

## 1. Introduction

Osteoarthritis (OA) is a progressive joint disease, most commonly observed in the middle-aged or elderly population, predominantly impacting women [1]. OA is a chronic and degenerative disorder affecting diarthrodial joints, involving the spine and peripheral joints, and specifically the hands, hips, knees, and feet [2]. OA presents an economic and social burden with a significant impact on patients’ quality of life, which is depicted by episodes of pain restricting everyday life and working activities [3].

The etiology of OA is still unclear, with investigations reporting a more complex disease mechanism which extends beyond a simple cartilaginous or bone disorder. OA notably involves several joint structures, including cartilage degeneration, abnormal bone remodeling, and synovial inflammation [4]. Moreover, Jiang et al., in a recent narrative review, described how the infrapatellar fat pad may play a crucial role in knee OA pain due to a crosstalk with the joint and the secretion of large amounts of inflammatory cytokines in OA disease [5].

OA is initiated due to a divergence between the mechanisms of cartilage deterioration and regeneration [6]. However, the onset of the disorder and the clear event sequence regarding the mechanism has been widely discussed in the literature. One proposed hypothesis posits that the secretion of pro-inflammatory cytokines within the synovial joint provokes matrix metalloproteinases, resulting in the fragmentation and degradation of the cartilage extracellular matrix, subsequently causing bone remodeling and synovitis [7,8]. In contrast, some investigations have indicated that subchondral bone remodeling and synovitis occur before articular degeneration in the early stages of OA [9,10]. On the other hand, in the later stages of OA, subchondral cysts, subchondral sclerosis, and osteophytes may form as a direct consequence of bone remodeling, cartilage degradation, and synovitis [11].

OA is a multifactorial disorder with similar biological and clinical features [12] which contribute to a restricted articular range of motion (ROM), as well as muscle fatigue and pain and overall escalation in disability, thereby exacerbating a decline in the quality of life. Radiographically, OA is identified by a chronic erosion of the articular cartilage, specifically involving the outermost layer [13]. This process is accompanied by subsequent subchondral sclerosis and the formation of cysts which contribute to the narrowing of the joint space, synovial inflammation, and the generation of marginal osteophytes. The aim of the present narrative review is to extensively describe all the treatments available in the current practice, further describing the most important innovative therapies.

## 2. Methods 

Two independent authors (LF. and FDF.) conducted a comprehensive search across multiple databases (PubMed, Web of Science, and Scopus). They reviewed each article’s title and abstract for studies available until December 2023. The search terms used were “(OA OR osteoarthritis) AND (pain) AND (treatment)”. The full texts of the studies were evaluated when eligibility could not be assessed from the title and abstract.

## 3. Relevant Sections

### 3.1. Pain and Disability in Osteoarthritis

Individuals affected with OA present with functional impairment and pain, which is considered a primary symptom of the disorder, occurring after physical activity and mitigated by rest during the early stages, persisting during intervals of sleep and rest at advanced stages. Additional symptoms of OA encompass stiffness following rest and joint instability [14]. Physical examination may reveal tenderness, crepitation, joint enlargement, deformity, and inflammation. Common findings in OA include restricted joint motion, muscle atrophy, muscle spasms, and flexion contractures [15]. At the functional level, patients may exhibit various disabilities depending on the affected joint and can be categorized into five groups: mobility, bending down, dexterity, bending the arm, and reaching up [16]. 

The origin of pain in osteoarthritis (OA) has not been thoroughly comprehended and is most effectively conceptualized within a biological, social, and psychological model which plays a fundamental role in the pain related to OA [14]. From a biological standpoint, neuronal activity may be considered crucial within the pain pathway in generating and exacerbating joint pain. Chemical mediators are released into the joint during the inflammatory phase, sensitizing primary afferent neurons [17]. As a result, apparently insignificant activities such as incremented physical exercise or donning high-heeled shoes or climatic changes will generate pain. Such neurophysiological response underlies allodynia, which is a sensation of pain following typically non-painful stimuli. In the long term, the elevated neurologic activity from the periphery may generate plasticity–distortion in the central nervous system through a mechanism known as “wind-up” [18]. An increase in the firing rate in the second-order neurons of the spinal cord may occur, enhancing the conveyance of nociceptive signals to the somatosensory cortex, thus amplifying the perception of pain and the presentation of pain stimuli within organisms that are distant from the inflamed joint, a phenomenon known as referred pain [19]. Inherent or intrinsic events may generate symptoms including self-efficacy or pain catastrophizing in patients, as well as the management of the social aspect of arthritis such as seeking social support and communicating the perception of pain [20].

### 3.2. Osteoarthritis Pain Management

Pain sensation is a key symptom of OA. In the early phases of OA, pain is related to activity and as the disease progresses, pain becomes chronic and is accompanied by intense pain attacks. The articular cartilage in adults is avascular and aneural [21]; therefore, the cartilage cannot directly generate pain or inflammation, particularly in the early stages of the disease before potential neurovascular invasion in late or end-stage disease [22]. Pathological alterations in non-cartilaginous joint tissues, such as the subchondral bone, synovium, joint capsule, periarticular ligaments, and periarticular muscle, are abundantly innervated and are potential sources of pain in OA [21,22]. During the inflammatory stage or the cartilage deterioration process, inflammatory mediators are discharged, sensitizing primary afferent nerves. The subchondral bone and pain receptors are thus exposed due to the stripped cartilage, leading to the vascular congestion of the subchondral bone and increased intraosseous pressure. Sensory nerve fibers have been reported in vascular channels in relation to osteochondral angiogenesis, suggesting a possible origin of symptomatic pain [23].

The management of OA primarily focuses on symptom relief, considering a limited understanding of the etiopathogenesis that hampers the development of appropriate disease-modifying drugs. According to the regulations established by the American College of Rheumatology (ACR) [24] and the Osteoarthritis Research Society International (OARSI) [25], the fundamental management of OA includes patient education and self-management, land-based exercises such as strengthening, cardio, balance, neuromuscular, or mind–body exercises, as well as weight management for overweight or obese individuals. Additionally, for knee OA, the guidelines suggest alternative management such as aquatic exercise, walking aids, topical and oral nonsteroidal anti-inflammatory drugs (NSAIDs), intra-articular steroid injections, and tibiofemoral bracing [24,25]. The OARSI guideline advocated for the use of topical NSAIDs but advise the use of intra-articular injections, oral NSAIDs, proton pump inhibitors, and COX-2 inhibitors only under specific conditions. In the case of hip OA, both guidelines strongly advise patient education, self-management, and land-based exercises as the key therapeutic strategies. The oral use of NSAIDs and mind–body exercises are highly recommended by both guidelines, while the ACR guidelines further suggest the use of walking aids, intra-articular glucocorticoid injections, and weight loss. For hand OA, the ACR and EULAR (European Alliance of Associations for Rheumatology) therapy counselling includes patient formation, hand exercises, and orthoses as the fundamental treatment approaches [26].

Presently, no treatment is able to modify the course of OA, and therapy is focused on mitigating pain and enhancing functionality. In particular, pharmacological medications are proposed which include nonsteroidal anti-inflammatory drugs (NSAIDs), acetaminophen, duloxetine, opioids, topical NSAIDs, and capsaicin [27]. Moreover, intra-articular injections commonly involve corticosteroids, hyaluronic acid (HA), ozone, plasma rich in growth factor, and platelet-rich plasma [28]. Clinical trials involving innovative intra-articular therapies are currently underway, including biological therapies, gene therapies, and cell therapies [29].

### 3.3. Intra-Articular Drug Delivery

Intra-articular (IA) injections offer direct access to the joint, with a potential to alleviate inflammatory symptoms. The prospect of IA injections is promising and advantageous in relation to systemic administration. However, a significant drawback lies in the prompt clearance of drugs from the joint, posing a substantial hurdle to the treatment efficacy [30]. At present, IA stands as the central component of non-surgical treatment for osteoarthritis (OA) [25]. Existing evidence indicates the effectiveness of the treatment in alleviating short-term pain in OA patients, enhancing joint function with minimal risk of patient injury [31]. IA injections allow for immediate access to the joint, thereby enhancing the targeted bioavailability of medications while reducing adverse events, overall exposure, and costs. Albeit the acknowledged safety of IA injections, the swift clearance of drugs imposes limitations on their therapeutic effects [32]. Additionally, factors such as systemic effects, administration techniques and drug residence time contribute to treatment variability [33].

### 3.4. Corticosteroid Injection

Corticosteroids exhibit anti-inflammatory and immunosuppressive potential, with a complex mechanism of action involving an immediate interaction with nuclear steroid receptors, disrupting both the immunologic cascade and the inflammatory process at various stages. Such a mechanism allows for corticosteroids to decrease vascular permeability and hinder the aggregation of inflammatory cells [27,28,30]. They also impede phagocytosis, the development of neutrophil superoxide, metalloprotease, and metalloprotease activator. Moreover, corticosteroids inhibit the synthesis and emission of numerous inflammatory mediators, including prostaglandins, as well as leukotrienes [34]. Five injectable corticosteroids have received approval from the Food and Drug Administration (FDA) for intra-articular (IA) injections which include methylprednisolone acetate, triamcinolone acetate, betamethasone acetate and betamethasone sodium phosphate, triamcinolone hexacetonide, and dexamethasone. In clinical practice, local anesthetics are frequently combined with corticosteroids prior to injection to provide rapid-onset analgesia. From this common practice there is no evidence to suggest that the addition of local anesthetics to corticosteroid preparations alters the physical properties or efficacy of the corticosteroid [35,36].

In the management of both acute and chronic inflammatory conditions, intra-articular corticosteroid injections are commonly employed [37]. These injections are particularly beneficial during osteoarthritis (OA) flares where indications of inflammation and joint effusion are present [38]. In this context, numerous meta-analyses have been conducted, investigating the optimal type of corticosteroid, assessing treatment safety, and, most importantly, evaluating its effectiveness in relation to pain relief duration. In a Cochrane systematic review [39], comparisons among various intra-articular corticosteroids revealed that triamcinolone hexacetonide demonstrated superiority over betamethasone in terms of reduction in pain lasting up to 4 weeks post-injection. Additionally, in other systematic literature reviews conducted in 2009, the action duration of intra-articular corticosteroid injections in alleviating pain was examined [40]. The findings of the review indicated the association of IA corticosteroids with a decrease in pain sensation that persisted for a minimum one-week period, suggesting that IA corticosteroids should be regarded solely as a transient treatment for chronic cases.

The subcommittee on osteoarthritis of the American College of Rheumatology (ACR) recommends corticosteroid injections as an effective method for reducing pain [24]. Similarly, the Osteoarthritis Research Society International (OARSI) advises the use of intra-articular corticosteroids for pain associated with symptomatic OA [25]. A systematic review indicates a modest therapeutic effect typically lasting for an average of 2 to 4 weeks [41]. Due to the restricted duration of therapy, booster injections (as many as four annual administrations) are commonly required. Recently, studies have reported the recurrent use of intra-articular corticosteroids in relation to an increase in cartilage loss [42]. Alternatively, the European Society for Clinical and Economic Aspects of Osteoporosis, Osteoarthritis and Musculoskeletal Diseases (ESCEO) has suggested a modest use of corticosteroids under specific conditions of contraindications to NSAIDs or inadequate alleviation following NSAID treatment, specifically for a reduced period of analgesia [43]. In contrast, the effectiveness of the corticosteroids injection presents uncertainties; thus in 2021, the European League Against Rheumatism (EULAR) proposed the use of intra-articular injection of corticosteroids only in cases of pain exacerbation, especially with accompanied effusion, avoiding the use of intra-articular steroids in cases of acute pain of a recent onset without an appropriate diagnosis [44]. However, the workgroup of the American Society of Orthopaedic Surgeons interpreted the evidence as inconclusive regarding the benefits of intra-articular corticosteroids, and refrained from providing administration recommendations in the guidelines for patients with symptomatic OA [45].

In summary, available research evidence suggests that intra-articular corticosteroid injections lead to a short-term reduction in pain associated with osteoarthritis. They may be considered an adjunctive therapy in conjunction with the core treatment for alleviating moderate-to-severe pain in individuals with OA.

### 3.5. Hyaluronic Acid Injection

Hyaluronic acid (HA) can be developed from harvested rooster combs or via in vitro bacterial fermentation [46]. HA is defined as a linear, non-sulphated glycosaminoglycan, formed primarily from repeating units of N-acetylglucosamine and glucuronic acid connected by β-(1-4) and β-(1-3) glycosidic bonds, contributing to an actively steadfast structure [47]. The disaccharides all possess a molecular weight (MW) of approximately 400 Da, and a hyaluronic acid chain may contain 10,000 disaccharides, resulting in a molecular weight of approximately 4.0 × 10^3^ kDa [48]. FDA-approved injectable hyaluronan products include sodium hyaluronate and high-molecular-weight hyaluronan [49].

Hyaluronan is a naturally derived extracellular matrix (ECM) molecule present in synovial fluid, playing an important role in joint lubrication. The altered characteristics of hyaluronan in the synovium may generate inflammation [46]. A reduced concentration and mean molecular weight of HA in the synovium are the consequences of diminished HA synthesis, increased HA degradation, and elevated oxidative stress [50]. Numerous investigations have reported that the exposure of chondrocytes and fibroblasts to low-molecular-weight (LMW) HA fragments (<400 kDa) may result in an upregulation of pro-inflammatory cytokines [7,51,52]. In contrast, high-molecular-weight (HMW) HA may provoke a contrasting effect on certain systems, restraining mediators such as TNF-α and IL-1β [53,54].

In normal synovial fluid, the HMWHA causes both joint lubrication and shock absorption. The viscoelastic activity of HA may vary according to the applied force. Under shear stress, HA serves as a lubricant which gradually decreases in viscosity, thereby permitting effortless mobility. Conversely, HA functions as a shock absorber under compression, preventing joint damage. Moreover, HMWHA in the synovial fluid is crucial for maintaining inherent joint integrity, which is required to protect synovial cells and regulate large molecule mobility within the joint, thereby avoiding free radical discharge, as well as preventing an inflammatory response [55]. In inflammatory contexts of osteoarthritis (OA) or rheumatoid arthritis (RA), HMWHA is dissipated by reactive oxygen species (ROS), reducing the viscous state and compromising its lubricating and shock absorbing potential [56]. This deterioration leads to impaired joint movement and pain [57]. Additionally, reductions in pain associated with intra-articular HA have been linked to a decreased development of bradykinin, prostaglandin E2, and substance P, along with the direct constraint of nociceptive afferents [58].

The intra-articular injection of HA may regenerate the regular viscoelastic features of morbidly modified synovial fluid, otherwise known as “viscosupplementation” [59]. It has been suggested that that HA temporarily reinstates the lubricating and shock absorbing potential of synovial fluid. Furthermore, previous investigations report the disease-modifying effects of viscosupplements [60], including a decrease in synovial inflammation [61], a protective effect in relation to cartilage erosion, and an enhancement of intra-articular HA production [62]. Specifically, in vitro experiments indicate that exogeneous low-weight HA administration can enhance the synthesis of extracellular matrix proteins, including chondroitin and keratin sulfate, and proteoglycans [62].

Hyaluronic acid possesses rheological properties which may alleviate pain through (i) the inhibition of the discharges of joint nociceptors, acting as an authentic elastoviscous filter; (ii) chemical hypersensitivity of nociceptive terminals within the inflamed joint tissues, most associated with the HA concentration [63]; (iii) and the coating of nociceptors located in the synovium and a potential molecular entrapment of pain signaling [64]. Moreover, single intra-articular injections of HA have been indicated in the reduction in pain perception due to the interference with lipopolysaccharide (LPS-triggered elevation) in the development of PGE2 and cyclooxygenase 2 (COX-2). Moreover, the activation of opioid receptors may generate the antinociceptive activity of HA [65].

HMWHA has the potential to inhibit the inflammatory events associated with osteoarthritis [48] by disrupting the actions of low-molecular-weight HA (LMW HA) fragments at CD44, receptor for HA-mediated motility (RHAMM), Toll-like receptors (TLR)-2, and TLR-4 [66]. In vitro investigations [67], as well as in vivo [68] studies, suggest that the administration of HMWHA results in a relevant anti-inflammatory response, which is partly mediated by the CD44 blockade. Moreover, exogenous HA reduces inflammatory cytokine and matrix metalloproteinase (MMP) levels in tissues extracted from patients affected by OA and other conditions in relation to joint injury [69], as well as the inhibition of IL-1β and TNF-α production [70].

Regarding OA, the American College of Rheumatology (ACR) subcommittee provides no specific recommendations about the use of intra-articular hyaluronates, and offers no indications regarding the various molecular weights of HA [24]. Furthermore, the OARSI fails to provide specifications about the molecular weights of HA that should be used to achieve a long-lasting effect.

It was also noted that intra-articular HA possessed a more adequate safety overview compared to frequent corticosteroid injection administration [25]. However, the American Society of Orthopaedic Surgeons discourages the use of IA hyaluronic acid in patients suffering from symptomatic OA of the knee due to absence of a minimum clinically important improvement (MCII) [71], although the guidelines state that HMWHA has shown superiority over LMWHA in the various studies analyzed. The workgroup highly considers the supporting evidence, as well as acknowledges the strength of the recommendation regarding the use of intra-articular HA in their guidelines. In Europe, the ESCEO provided a weak recommendation for HA, suggesting its use solely in cases of individual contraindication to NSAIDs or inadequate pain relief regarding NSAID therapy [43], with reports also of the inferiority of LMWHA and that HMWHA is, indeed, related to an elevated incidence of adverse events. In contrast, the EULAR recommends the use of HA injections in OA treatment, reporting the efficacy of HA in OA treatment to reduce pain and improve mobility [72].

In summary, the findings indicate the overall safety of intra-articular HA injections and potential efficacy in reducing pain in mild OA of the knee for a duration of 24 weeks. However, the expense of such treatments is of significant concern and patients are to be duly informed. Therefore, in addition to considering patient expectations, healthcare providers are required to assess the cost effectiveness before proposing the treatment.

### 3.6. Analgesics/Anti-Inflammatory Drugs

Non-steroidal anti-inflammatory drugs (NSAIDs) are frequently administered for the treatment of OA [73]. NSAIDs represent a large class of compounds classified according to their chemical structure, target selectivity, and pharmacokinetic characteristics. NSAIDs include salicylic acid derivatives such as aspirin, aryl acetic acid derivatives such as ibuprofen and naproxen, indole acetic acid derivatives such as indomethacin, anthranilic acid derivatives such as diclofenac, and enolic acid derivatives such as meloxicam [74]. NSAIDs prevent the conversion of arachidonic acid into prostanoids, including prostaglandin, prostacyclin, and thromboxane, by inhibiting the cyclooxygenase pathway. NSAIDs interfere with both isoforms of cyclooxygenase (COX-1, COX-2); however, the degree of inhibition will vary according to the selectivity of the NSAID. Frequently employed NSAIDs such as diclofenac, naproxen, ibuprofen, and aspirin non-selectively restrain both COX-1 and COX-2. In contrast, celecoxib and meloxicam are able to inhibit both COX enzymes, with preferential selectivity for COX-2 [73,74]. COX-2 inhibitors reduce prostacyclin formation in favor of thromboxane, a prothrombotic eicosanoid. The relative increase in thromboxane, coupled with a diminution in prostacyclin, can lead to the development of thrombotic cardiovascular events [58]. On the other hand, a nonselective COX-1 and COX-2 inhibitor may result in gastric toxicity due to the inhibition of COX-1 in gastric mucosa [72]. While the oral administration of NSAIDs is the most common route, intra-articular injections of NSAIDs have gained popularity in various settings, with the aim of potentially increasing NSAID concentration in the target tissue and, simultaneously, reducing the systemic complications associated with the use of NSAIDs [58,72]. Selig DJ and colleagues [74] reviewed the pharmacokinetics, safety, and efficacy of intra-articular NSAID injections, analyzing both animal and human studies reporting on the safety and effectiveness of single doses of intra-articular NSAIDs. The analysis of different studies indicates that single doses of intra-articular NSAIDs result in significantly lower whole systemic and synovial exposure in relation to a one-week administration of oral NSAIDs. However, the peak concentrations obtained in the synovium following intra-articular administration were notably greater. The safety profile of intra-articular NSAIDs was shown to be optimal. In human studies, intra-articular NSAIDs were as efficient as orally administered NSAIDs and intra-articular corticosteroids in alleviating pain in relation to OA. However, to the best of our knowledge, no approved NSAID has currently been identified on behalf of the Food and Drug Administration (FDA) for intra-articular injection. Opioids (i.e., tramadol) are effective for the management of acute musculoskeletal pain, although there is little evidence specific to their use for OA pain [27].

Over the last 20 years, several studies have investigated the ability of glucosamine sulfate to improve the symptoms (pain and function) and delay the structural progression of osteoarthritis. There is now a large, convergent body of evidence that glucosamine sulfate, given at a daily oral dose of 1500 mg, is able to significantly reduce the symptoms of osteoarthritis in the lower limbs [75].

### 3.7. Platelet-Rich Plasma

Platelet-rich plasma (PRP) is characterized by a segment of the liquid phase of autologous blood containing a greater concentration of platelets [76]. PRP therapy has been adopted for numerous conditions for three decades, gaining remarkable attention in regenerative medicine [77]. The platelet concentrate is activated through the addition of calcium chloride, forming a platelet gel and the discharge of growth factors and bioactive molecules [78]. Consequently, platelets play an active role in the healing mechanism through a diverse range of growth factors and other active molecules to the injured site. Several issues in relation to platelet-rich plasma (PRP) in a joint with OA remain debatable [79], along with the use of PRP in relation to decreased inflammation [80], pain alleviation [81], improved functionality [82], and potential cartilage redevelopment [83]. The main challenge lies in understanding the complex mechanisms concerning the potential therapeutic effects of platelet-rich plasma (PRP), primarily attributable to the diverse study methodologies which commonly imply a high risk of bias. Following the findings of the pioneering study by Sampson S. and Colleagues [84], numerous investigations have explored the impact of platelet-rich plasma (PRP) in treating OA. Overall, the findings consistently indicate that PRP is a safe and cost-effective treatment modality [85], offering sustained symptomatic and functional improvement [86]. The therapeutic effect of platelet-rich plasma (PRP) is currently associated with multiple factors, including its preparation, formulation, and the frequency of injections, as discussed in various studies [87]. Controversy surrounds the optimal proposal for PRP application due to divergent findings in various studies [88,89].

Notably, standardized guidelines for PRP preparation were lacking in the literature during the trial process. Despite general recommendations supporting the use of intra-articular PRP injections for OA patients, it is important to note that the FDA has not granted approval for this therapeutic intervention [90].

The ACR and OARSI guidelines strongly advise against the use of PRP until clarification is addressed in further studies, concerning also the lack of variability in and absence of standard procedures regarding accessible PRP preparations and techniques [25]. The AAOS guidelines refrain from stating specific recommendation in the use of PRP [91], and the ESCEO fails to include PRP in its guidelines [43]. In the treatment of knee OA, PRP lacks recommendations due to a lack of evidence, as outlined in the EULAR guidelines [72]. In the light of ample patient demand regarding the biologic treatment of orthopedic disorders, the American Academy of Orthopaedic Surgeons organized a coordinated symposium and established a common framework [92] to enhance and expedite the clinical assessment, utilization, and improved application of biologic treatment for musculoskeletal diseases. Alongside recommendations for the initiation of high-quality multicenter clinical trials, physicians and institutions are highly recommended to advocate for biologic therapies to establish comprehensive patient databases which may be linked to biorepositories, enabling post-market surveillance and quality assessments. In vitro studies have reported that PRP has the ability to promote the chondrogenic differentiation of stem cells [77]. When PRP and stem cells are co-transplanted into the knee joint, a variety of growth factors can stimulate and induce stem cells to differentiate into chondrocytes, and then promote the recovery of cartilage injury. However, whether the effect of PRP injected into human joints can play the same role needs further clinical experimental research.

In summary, studies suggest that PRP holds promise in alleviating pain, enhancing knee functionality, as well as improving the quality of life. Nevertheless, data are lacking in the indications of PRP to regress osteophytes or regarding its ability to promote the regeneration of cartilage. More favorable outcomes are observed in younger patients and in cases of mild OA. Intriguing preliminary outcomes and the growing clinical administration of this therapeutic approach are evident, but the extensive use of PRP in OA lacks the robust support of high-quality evidence to demonstrate unequivocal clinical improvement.

### 3.8. Mesenchymal Stem Cell Therapy

The use of adipose tissue in treating orthopedic conditions has been closely investigated recently [29,91,92,93,94]. Adipose-derived stromal or adipose stem cells (ASCs), classified as mesenchymal stem cells, are derived from adipose tissue situated in the perivascular microcirculation within adipose tissue [93,95]. Research indicates that, when compared to bone marrow (BM), ASCs exhibit a higher numerical presence per unit volume, a higher proliferative ability, and higher immunomodulatory capacity [95]. In the past, ASCs were isolated using digestive enzymes from stromal vascular fraction suspensions. This approach posed challenges, not only due to the intricate harvesting processes involved, but also as a result of regulatory apprehensions regarding the manipulation of cells and cell expansion [96]. The exposure of adipose tissue to collagenase has been regarded as more than “minimally manipulated”. The term “minimally manipulated” is described by the U.S. Food and Drug Administration recommendations as “processing that does not alter the original relevant biological characteristics of cells” [97]. As a result, numerous efforts have been undertaken to devise collagen-free techniques in the isolation of stromal/vascular cells, and several of these methods have been patented [91,98]. This approach yields ready-to-use stromal vascular fraction (SVF) or SVF-supplemented fat, eliminating the need for enzymatic processes. SVF may be described as a heterogeneous compilation of cell varieties derived from the enzymatic or manual digestion of lipoaspirates. It encompasses various cell types such as endothelial progenitors, hematopoietic cells, endothelial cells, adipose-derived stromal/stem cells (ASCs), adipose progenitors pericytes, immune cells, fibroblasts, macrophages, leukocyte subtypes, lymphatic cells, and smooth muscle cells [99,100]. Furthermore, the presence of pericytes within the SVF is noteworthy, as these cells can differentiate into active mesenchymal stem cells (MSCs) in reaction to inflammation and injury, playing a key role in the regeneration mechanisms [101,102]. The therapeutic potential of MSCs is demonstrated through two primary aspects: the regeneration of injured tissue and relevant immunomodulatory activity [103]. The pleiotropic impact acts as a focal point in MSC therapy, facilitating the discharge of various soluble factors that exert antioxidant, immunomodulatory, antiapoptotic, and angiogenic effects. The regenerative and immunomodulatory responses of MSCs are activated via paracrine activity, which implies the secretion of proteins/peptides as well as hormones [104]. Additionally, an anti-inflammatory effect has been observed through the release of exosomes containing various microRNAs (miRNAs), promoting cellular proliferation during the regeneration of bone tissue [105].

MSCs have garnered significant attention in the treatment of OA. However, the specific efficacy of stem cell treatment in treating OA has yet to be clarified. In recent years, several studies have reported the short duration and mid-duration of stromal vascular fraction (SVF) for OA, demonstrating improvements in joint function and analgesic effects. In a recent meta-analysis conducted by Muthu S. and colleagues [106] on 21 publications, the authors highlighted the reduction in the VAS score in patients with knee osteoarthritis treated with SVF from adipose tissue, with seven studies reporting a statistically significant improvement at 6 months after treatment, while all other studies resulted in a statistical improvement for flow-up periods between twelve and twenty-four months. Issa MR. and colleagues [107] conducted a meta-analysis on four randomized controlled trials, observing a statistically significant decrease in the Western Ontario and McMaster Universities Osteoarthritis Index (WOMAC) score in the SVF group compared with the control group after 6 months, which remained stable even after 12 months. Moreover, they showed a statistically significant reduction in the pain score (VAS, NRS) in the SVF group compared with the control after 6 months. A corresponding and statistically significant reduction was also observed after 12 months. Finally, the authors examined the quality of life and functionality using Knee Injury and Osteoarthritis Outcome scores (KOOS), and found a statistically significant reduction in the SVF group compared with the control after a period of 6 and 12 months. In another meta-analysis on 18 publications conducted by Agarwal N and colleagues [108], the authors highlighted improvement in the WOMAC score in patients with knee osteoarthritis treated with SVF from adipose tissue, and highlighted the four studies reporting a statistically significant improvement within one month after treatment, while all other studies resulted in a statistical improvement for flow-up periods between two and twenty-four months. Furthermore, Kim K. and colleagues [109] performed a phase 3, randomized, double-blind, placebo-controlled trial on 125 knee osteoarthritis patients and observed significantly improved outcomes in 100 mm VAS and total WOMAC score compared with the control group after 6 months. The radiologic outcomes and adverse events revealed no significant differences between the groups. No serious treatment-related adverse events were observed. Concerning osteoarthritis of the trapeziometacarpal joint, a meta-analysis [110] examined seven human-based studies. Notably, no randomized controlled trials were conducted. Across all studies, the minimal processing of the collected adipose tissue involved centrifugation or mechanical homogenization, either alone or combined with filtration and decantation. None of the investigations specified the extraction of the stromal vascular fraction using enzymatic or mechanical methods, nor did they address the examination of the injected tissue. While all studies evaluated pain appraisal before and after treatment using the visual analogue scale (VAS), it is noteworthy that two different scales were utilized (1–10 or 1–100), along with different methodologies for grip strength measurement. Due to these discrepancies, no statistically significant correlation was established. In cases involving the knee, advanced, controlled, and randomized studies were performed; however, the current body of evidence for trapeziometacarpal joint osteoarthritis lacks effectiveness, indicating a crucial need for further research in this area.

Nevertheless, the use of MSCs has been strongly discouraged by the American College of Rheumatology (ACR), the Arthritis Foundation (AF), and the Osteoarthritis Research Society International (OARSI) [24,25]. This recommendation is attributed to the diverse methodologies employed in clinical studies, including discrepancies in the tissue origins of MSCs, variations in cell numbers, and differences in culture methods. Such variations in application strategies may impact the therapeutic effects and consequently influence clinical responses. It is noteworthy that MSC therapy was not incorporated into the guidelines provided by the American Academy of Orthopaedic Surgeons (AAOS) [71], European Society for Clinical and Economic Aspects of Osteoporosis, Osteoarthritis, and Musculoskeletal Diseases (ESCEO) [43], and the EULAR [26,72].

To date, bone marrow mesenchymal stem cells (BM-MSCs) are considered the gold standard of bone and cartilage tissue engineering [111,112]. These cells can migrate from subchondral bone to damaged sites, differentiate into chondrocytes and osteoblasts, and integrate new tissues and surrounding tissues, thus probably playing an important role in the repair of bone and cartilage. MSCs can protect cartilage by differentiation into chondrocyte lineages, affecting the chondrocytes, mediating mitochondrial function, regulating cytokines, balancing the synthesis and catabolism of the extracellular matrix (ECM), modifying immune reactions, and paracrine activity that might be involved with the secreted exosomes.

A recent network analysis reported that compared with the placebo, both BM-MSCs and ASCs could provide pain relief and improve knee function during a 6-month follow-up period. Nonetheless, at a 12-month follow up, only ASCs had a potential clinical therapeutic effect on pain relief [113]. At present, several studies believe that ASCs are more diverse and have more abundant sources than BM-MSCs. Therefore, for elderly patients with OA, ASCs are more effective [114].

### 3.9. Innovation in Osteoarthritis Therapy

Nanotechnology finds widespread application across diverse industries and in the field of medicine, including clinical applications in the enhancement of targeted delivery regarding both conventional and biological drugs. At present, nanoparticles (NPs) stand out as cutting-edge biomaterials with significant potential for diagnosing and managing OA [115]. Nanomaterials, including liposomes, micelles, carbon nanoallotropes, and quantum dots, are characterized by sizes within the range of 1–100 nm [116]. Recently, research has reported the crucial purpose of nanotechnology in treating OA through the development of drug delivery approaches. These systems have proven to enhance specific targeting, improve drug delivery ability, amplify the therapeutic response, minimize adverse events and prolong medication adherence, as well as improve circulation time, and impede the dispersion and drug decomposition in body fluids [117,118]. A surge of interest has occurred regarding the clinical application of exosomes derived from MSCs, typically isolated from the synovium, adipose tissue, and bone marrow [119]. Studies have unveiled that exosomes derived from MSCs exhibit the potential to safeguard cartilage and bone from degradation in OA [120], achieved through mechanisms such as upregulating the expression of chondrocyte markers such as type II collagen and aggrecan, downregulating catabolic markers, reducing inflammatory markers (iNOS), safeguarding chondrocytes from apoptosis, and inhibiting macrophage activation.

Therefore, exosomes derived from MSCs exhibit the ability to regenerate joint components using two distinct secretion functions: (a) anti-inflammatory events through the MSC downregulation of inflammatory signals in osteoarthritic cartilage by secreting interleukin (IL)-1β, IL-6, IL-8, matrix metalloproteinase (MMP)-1, and MMP-13 [121]; and (b) trophic events, whereby molecules induce cell proliferation, reduce adherent tissue formation, and enhance the regeneration of endogenous cartilage. Such mechanisms may involve the epithelial growth factor (EGF), insulin-like growth factor (IGF)-1, basic fibroblast growth factor (bFGF), transforming growth factor (TGF)-β, and vascular endothelial growth factor (VEGF) [122,123].

Innovative studies from animal models reported on the use of microgels and hydrogels with a microencapsulation of stem cells in order to protect the injected cells, prolonging in vivo retention and widening the therapeutic window [124]. Jonhbosco et al. demonstrated that the injection of microencapsulated MSC in OA rat joints showed a significant reduction in cartilage degradation and matrix loss [125]. Furthermore, even more recently, Ma et al. developed a nanoparticle-based formulation of pazopanib, an FDA-approved anticancer drug that targets both VEGFR1 and VEGFR2. The authors demonstrated that a single intra-articular injection of it was able to effectively reduce joint pain for a prolonged time without substantial side effects in preclinical OA mouse models [126]. Other studies pointed out the role of NGF/TrKA signaling in OA pain. It has been demonstrated that NGF blocking was also effective in relieving OA pain. However, these studies have also elicited concerns about major side effects including osteonecrosis (ON) and rapidly progressive OA (RPOA). New research has deepened new treatment options such as Wnt inhibitors, TNF, and IL-1Beta antibodies, but further studies are needed for their use in clinical practice [111].

## 4. Future Directions

Scientific research is exploring various alternatives for OA pain management, including tissue regeneration techniques [112], personalized medicine [127], and targeted drug therapies [111]. Advancements in understanding the molecular and genetic aspects of osteoarthritis may lead to more effective and tailored treatment approaches in the future.

OARSI and ESCEO defined recommendations in proposing two algorithms to provide information regarding the non-surgical treatment of knee osteoarthritis [128], yielding evidence-based and expert-reviewed advice to healthcare providers. The outcomes offer comparable information concerning fundamental treatments, and both guidelines offer analogous, evolving management algorithms, albeit with some distinctions, especially in the sequencing of treatments regarding therapy. Both algorithms strive to customize the therapeutic protocol based on patient characteristics, a crucial aspect when contemplating the administration of oral NSAIDs and COX2-selective inhibitors. Indeed, several studies have reported that several cytokines such as IL-1β, IL-6, TNF-α, and NGF, and chemokines could be released into the joint fluid, causing the development of pain and an increased sensitivity of peripheral nerves resulting from interaction with sensory nerves [129,130,131]. Previous pre-clinical studies reported an observed elevation in the expression of nerve growth factor (NGF) in the articular cartilage, serum, and synovial fluid in cases of OA [132,133]. Moreover, new insights regarding the cellular mechanism of OA physiopathology have led to the development of emerging pharmacological therapies. NGF/TrkA signaling inhibitor, TNF and IL-1Beta antibody, Wnt inhibitor and ion channel modulator have been analyzed in human clinical trials from phase 2 to phase 4 [111]. Nevertheless, further studies are required for clinical applications due to adverse occurrences such as a raid and pronounced deterioration of the joints. In recent years, several studies have highlighted the role of synovial mast cells in knee osteoarthritis [134,135]. The existence of mast cell-derived factors, including histamine, in the synovial fluid of OA patients implies initiation and engagement in the disease mechanism [136]. For these reasons, the inhibition of degranulation of mast cells and/or inhibition of their activation pathways may pave the way for new disease-modifying treatments for OA, targeting the multiple functions of MCs [137,138].

## 5. Conclusions

Therapeutic interventions currently fail to offer adequate pain alleviation, with persistent use of drugs causing important adverse effects and toxic responses. Generally, an inconsistency among guidelines can be noted regarding the recommended pharmacological and non-pharmacological interventions. The decision-making process is common, based on the physician who is required to navigate through numerous molecules, physical therapies, and rehabilitation protocols. Table 1 reports our protocol for managing pain osteoarthritis.

Hyaluronic acid and corticosteroids are widely used agents, delivered through intra-articular injection to enhance joint lubrication and manage pain. The efficacy of intra-articular therapies, including HA and corticosteroids, is constrained by their rapid clearance; therefore, secure therapeutic preparations that offer sustained and prolonged drug release are mandatory. To achieve this goal, various materials, including synthetic products and natural (bio) materials, have been employed to obtain favorable outcomes, such as prolonged duration of articular retention, along with a gradual and controlled drug release, and the biodegradation of drug delivery carriers. It is imperative to highlight that osteoarthritis is characterized by a variety of morbidities according to different stages of disease development and it is evident that the pathogenesis of OA is encompassed by complexity and diversity; thus, there is a need to transcend the concept of “one cure fits all” treatment toward the development of tailored therapies. Studies are underway to develop different molecular-level mechanisms to explain the variety of symptoms and the mechanisms related to OA. Guidelines and protocols are thus imperative to establish “personalized” OA treatments, as well as the necessity for biomarkers which will facilitate the early diagnosis of OA, enabling early treatment from symptom onset, as well as ensuring a more favorable and long-term outcome.

## Figures and Tables

**Table 1:** Authors’ protocol for the treatment of OA.

Type	Injectate	Recommended Dose for Major Joints	Indications	References
Corticosteroid	Lidocaine + Methylprednisolone acetate	10 mg + 40 mg (1 mL total volume)	Night pain, knee swelling, prior to HA	Arroll B et al., 2004 [35] Estee M et al., (2022) [36]
	Triamcinolone acetate	40 mg (1 mL total volume)		
	Methylprednisolone acetate	40 mg (1 mL total volume)		
Glycosaminoglycan	Hyaluronic acid	2 cc total volume 2% concentration 3 infiltrations (1 every 15 days)	Pain due to physical activity, joint cracking	Bellamy N et al., 2006 [39] Fallacara A et al., 2018 [47]
Analgesics/Anti-inflammatory drug	Etoricoxib	90 mg 1 cp/die × 5 days followed by 1 cp/die each 3 days for 2 weeks	Useful in combination of infiltration therapy	Stoll V et al., 2023 [73]
	Acetaminophene	1 g max 3 times by day		
Autologous compounds	PRP	3 infiltrations in 3 weeks 2 cc volume	Useful after failed HA infiltrations	Everts P et al., 2020 [77] Gato-Calvo L et al., 2019 [79]
Mesenchymal stem cells	Stromal Vascular Fraction (SVF)	1 infiltration 2 cc total volume	Third-line of treatment after HA and PRP	Screpis et al., 2022 [96] De Francesco et al., 2021 [29]
